# Successful combination strategy of preoperative placement of an endoscopic nasopancreatic drainage catheter and pancreas preservation surgery for pancreatic injury with major pancreatic duct disruption: a case report

**DOI:** 10.1186/s40792-019-0743-1

**Published:** 2019-11-21

**Authors:** Kenji Kandori, Wataru Ishii, Ryoji Iizuka

**Affiliations:** Department of Emergency and Critical Care Medicine, Japanese Red Cross Society Kyoto Daini Hospital, 355-5 Haruobicho Kamigyoku, Kyoto, 602-8026 Japan

**Keywords:** Endoscopic nasopancreatic drainage (ENPD), Endoscopic retrograde pancreatography (ERP), Major pancreatic duct (MPD), Pancreatic injury, Pancreas preservation, Trauma

## Abstract

**Background:**

The guidelines recommend pancreatic resection for grade III and IV pancreatic injuries. On the other hand, organ preservation is an important issue. Herein, we present the first case of pancreatic injury with major pancreatic duct (MPD) disruption that was treated with the combination of preoperative placement of endoscopic nasopancreatic drainage (ENPD) catheter and pancreas preservation surgery after endoscopic pancreatic stenting (EPS) failure.

**Case presentation:**

A 70-year-old female diagnosed with pancreatic injury was admitted to our hospital. She was hemodynamically stable. ERP revealed MPD disruption, and EPS failed. An ENPD catheter was placed preoperatively at the site of injury. During laparotomy, we identified a partial-thickness laceration in the pancreatic body. At the site of injury, the tip of the ENPD catheter was found; therefore, the patient was diagnosed with grade III pancreatic body injury with MPD disruption. The extent of crush was not severe, and we had no difficulty in identifying the distal MPD segment. We inserted the ENPD catheter into the distal MPD segment. The ruptured MPD and the laceration was sutured, then pancreatic resection was prevented. She was discharged on POD 56.

**Conclusion:**

The treatment strategy incorporated ERP, placement of an ENPD catheter preoperatively, and a simple surgery in a hemodynamically stable patient with pancreatic injury allows the pancreas and spleen to be preserved.

## Introduction

Pancreatic trauma is a rare type of injury, which accounts for 0.2–0.3% of all traumas [1], and it is characterized by high morbidity and mortality. The American Association for the Surgery of Trauma (AAST) scale for pancreatic injuries [2] is the most common grading system. For grade I and II injuries, the guideline recommends non-operative or non-resectional management. Meanwhile, for grade III and IV injuries, pancreatic resection is recommended [3]. By contrast, based on the data obtained from a systematic review, early endoscopic retrograde pancreatography (ERP) and ductal stenting may treat the injured duct in some cases, even for grade III injuries; thereby, performing major laparotomy and resection is prevented [4]. It is important for preservation of pancreatic function to prevent pancreatic resection.

Herein, we present a case of AAST grade III pancreatic injury that was successfully treated with the combination of ERP, preoperative placement of an endoscopic nasopancreatic drainage (ENPD) catheter at the ruptured site, and pancreas preservation surgery.

## Case presentation

**Fig. 1:**
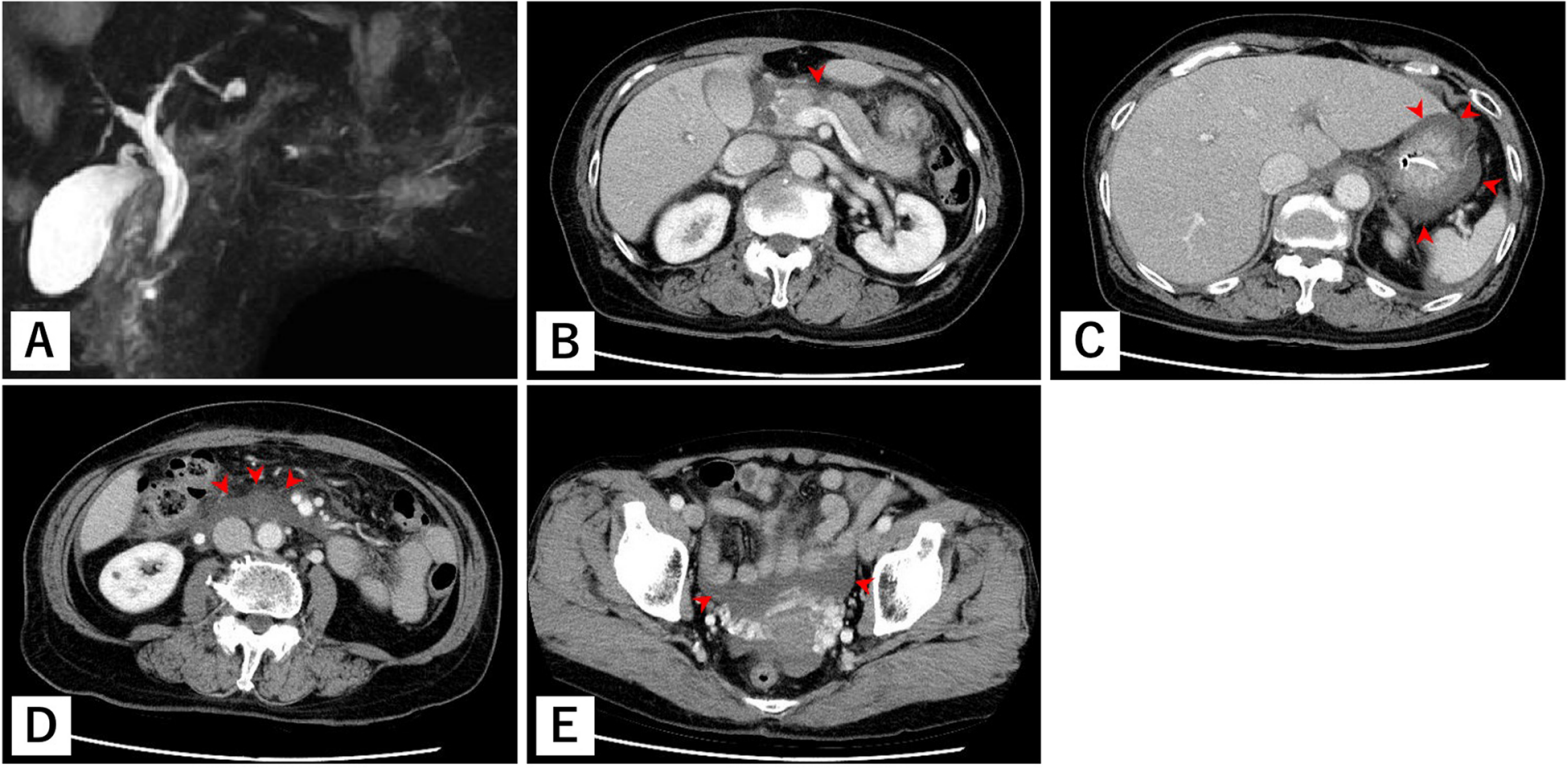
Preoperative MRCP and CT findings. From MRCP, MPD disruption was unidentified (**a**). The CT scan revealed pancreatic injury (**b**) and increase in fluid collection around the stomach (**c**), the pancreatic injury site, retroperitoneum (**d**), and pelvic cavity (**e**)

A 70-year-old woman was previously admitted in another hospital due to injury after falling onto her bicycle handlebars. Enhanced computed tomography (CT) scan revealed injury in the pancreatic body; thus, she was transferred to our hospital for further treatment 3 h after injury. We could not identify the presence of major pancreatic duct (MPD) disruption based on a previous CT scan. MPD disruption was not also identified on magnetic resonance cholangiopancreatography (MRCP) (Fig. 1a). She was hemodynamically stable since admission.

**Fig. 2:**
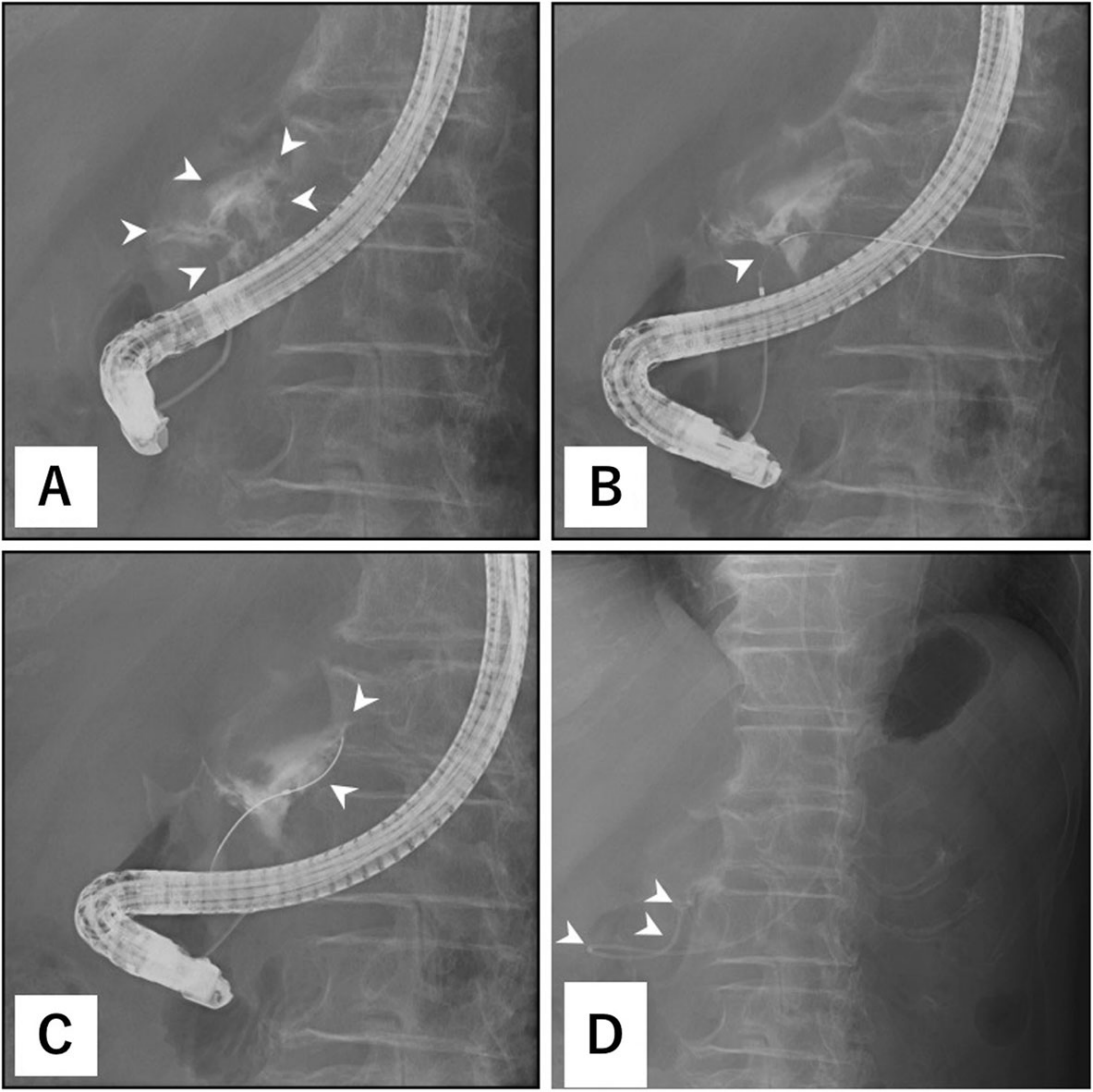
Preoperative endoscopic retrograde pancreatography and placement of an endoscopic nasopancreatic drainage catheter. Endoscopic retrograde pancreatography showed leakage of contrast medium (arrowhead) from the injured major pancreatic duct, and the pancreatic tail could not be visualized (**a**). A guidewire was passed across the break (arrowhead) to the distal pancreatic duct (**b**); however, the endoscopic nasopancreatic drainage (ENPD) catheter was not advanced across the rupture because the guidewire and catheter were warped (arrowhead) at the injury site (**c**). A 5-French ENPD catheter was placed at the injury site (arrowhead) preoperatively (**d**)

On the following day, CT scan was again performed and showed an increase in fluid around the pancreatic injury site, retroperitoneum, and pelvic cavity (Fig. 1b–e). Laboratory data showed elevated serum amylase and lipase levels (Table 1). Thus, we performed ERP and identified leakage of contrast medium from the injured MPD. Moreover, the pancreatic tail could not be visualized (Fig. 2a). A guidewire was passed across the break to the distal pancreatic duct (Fig. 2b); however, the ENPD catheter was not advanced across the rupture because the guidewire and catheter were warped at the site of injury (Fig. 2c). We decided to perform laparotomy following after ERP. A 5-French ENPD catheter was placed at the site of injury preoperatively (Fig. 2d).

**Table 1:** Preoperative laboratory investigations

White blood cells	6300/μL
Hemoglobin	11.8 g/dL
Platelets	210,000/μL
Albumin	3.5 g/dL
Total bilirubin	1.7 mg/dL
Leucyl aminopeptidase (LAP)	46 U/L
Alkaline phosphatase (ALP)	182 U/L
γ-Glutamyl transpeptidase (γ-GTP)	17 U/L
Aspartate aminotransferase (AST)	27 U/L
Alanine transaminase (ALT)	20 U/L
Lactate dehydrogenase (LDH)	252 U/L
Creatine kinase (CPK)	137 U/L
Cholinesterase (ChE)	237 U/L
Amylase	1100 U/L
Lipase	1969 U/L
Blood urea nitrogen (BUN)	11.2 mg/dL
Creatinine	0.47 mg/dL
C-reactive protein (CRP)	4.22 mg/dL
Prothrombin time (PT)	93.6%
Activated partial thromboplastin time (APTT)	27.0 s
Fibrinogen	384 mg/dL
D-dimer	4.4 μg/mL

**Fig. 3:**
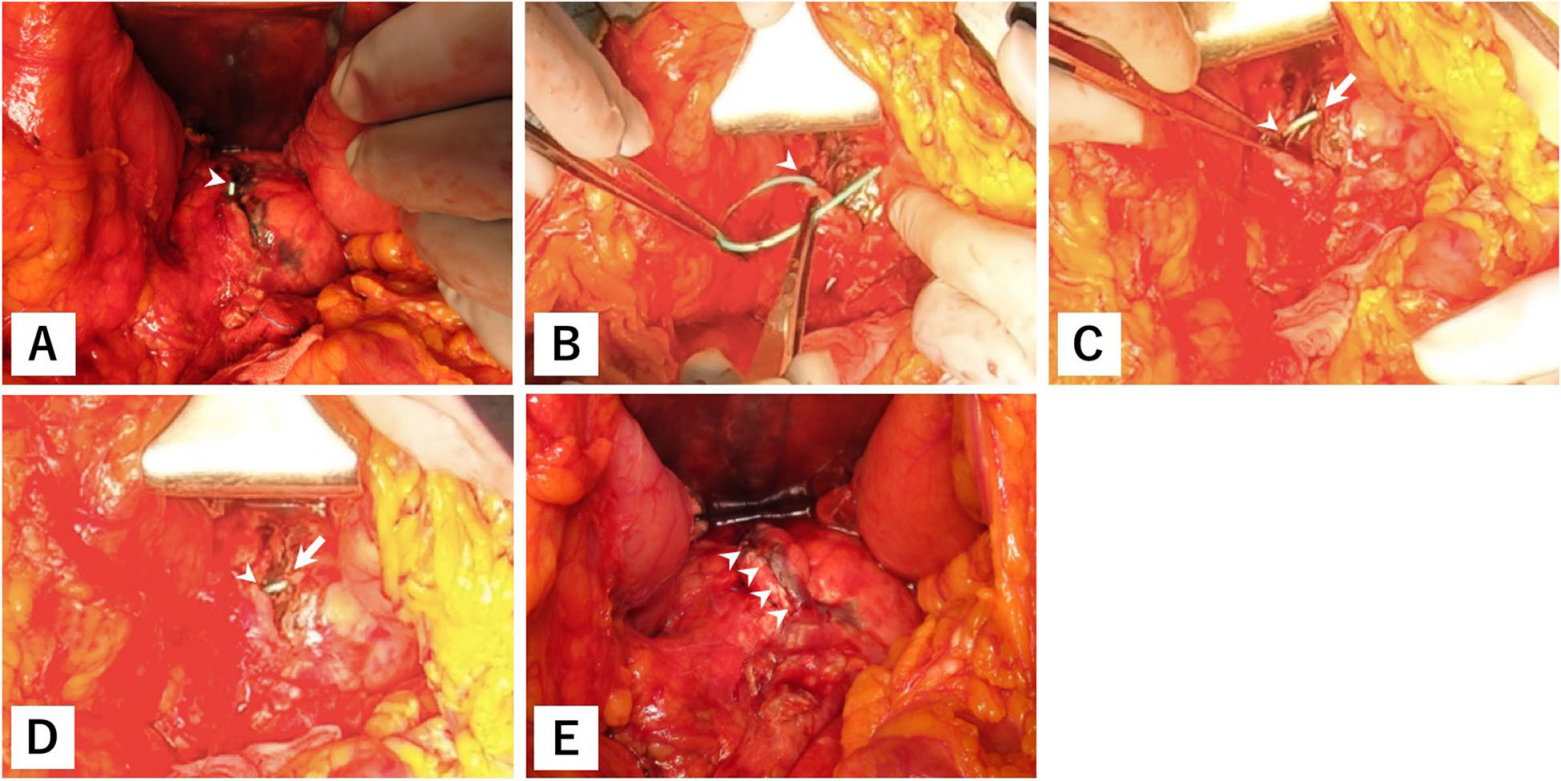
Operative findings. A partial thickness laceration in the pancreatic body and the tip of the endoscopic nasopancreatic drainage (ENPD) catheter (arrowhead) was identified (**a**). We inserted the ENPD catheter into the distal (arrow) major pancreatic duct (MPD) segment from the proximal (arrowhead) MPD segment (**b**–**d**). The raptured MPD and laceration (arrowhead) were sutured (**e**)

Surgery was started 22 h after injury and 2 h after ERP. During laparotomy, we found moderate amount of bloody ascitic fluid and saponification. We identified a partial thickness laceration in the pancreatic body. At the site of injury, the tip of the ENPD catheter was found (Fig. 3a), and we immediately identified the proximal MPD segment. Therefore, the patient was diagnosed with grade III pancreatic body injury with MPD disruption. The extent of crush was not severe, and we experienced no difficulty in identifying the distal MPD segment. We pulled the ENPD catheter out of the proximal MPD segment and inserted it to the distal MPD segment (Fig. 3b–d). After obtaining a deflection of the ENPD catheter, the ruptured MPD was repaired with multiple interrupted sutures. The laceration was sutured with multiple horizontal mattress sutures (Fig. 3e). In total, the patient lost 525 mL of blood. Blood transfusion was not required, and the operative time was 139 min.

The postoperative course was uneventful. On postoperative day (POD) 31, we performed ERP, and it showed no leakage of contrast medium from the MPD, and this result indicates that the patient’s injury was treated. The ENPD catheter was changed to the endoscopic pancreatic stenting (EPS) catheter using a guidewire. She was ambulatory and was then discharged on POD 56. The EPS catheter will be placed for 6 months.

## Discussion

The current case presented two important clinical issues. First, in the case of EPS failure, preoperative placement of an ENPD catheter is a promising strategy for definitive surgery of pancreatic injury with MPD disruption, and such procedure may allow the surgery to be much simpler and the pancreas and spleen to be preserved. Second, ENPD catheter inserted preoperatively is effective during and after surgery.

First of all, when EPS fails, preoperative placement of an ENPD catheter helps in the definitive surgery of pancreatic injury with MPD disruption, and such procedure enables simpler surgery and pancreas preservation. Several reports have presented pancreatic injury treated with EPS; however, this was the first case of pancreatic injury that was successfully treated and preserved the pancreas with the combination of preoperative placement of an ENPD catheter and surgery. In our case, treatment with EPS failed; thus, an ENPD catheter was placed at the injury site preoperatively. ENPD helped identify the proximal MPD segment. The location of the distal MPD segment from the proximal MPD segment was roughly estimated and easily identified because the degree of crush in the pancreas was not as severe. In case the crush is severe, debridement of the distal edge of the pancreatic body may help identify the distal MPD segment. In cases when pancreatic resection is inevitable, the operative time will be shorter if the proximal MPD segment is immediately identified. We successfully inserted an ENPD catheter into the distal MPD segment, which is a simple procedure. In trauma surgery, it is important to choose a simple technique, and our strategy is suitable for patients with pancreatic injury who have a hemodynamically stable condition.

In addition, it is important to mention that we could achieve organ preservation: the pancreas and spleen. It is expected that pancreatic endocrine function will be impaired in pancreatic surgery. Wittingen et al. showed that the islet concentration in the tail of the pancreas is significantly greater than in the head or body [5]. Slezak et al. reported the effects of pancreatic resection on glucose metabolism, and among distal (40–80%) pancreatectomy group, 17% had diabetes preoperatively, which increased postoperatively to 32% [6]. Jones et al. reported that 37% of patients who underwent distal resection of at least 80% developed diabetes [7]. In trauma surgery, it is important to consider pancreatic resection because it is safe to manage pancreatic injury with the first priority of lifesaving; however, we should also consider the possibility that pancreatic resection may reduce the quality of life in long-term survival. In that point, our treatment strategy which can prevent pancreatic resection is reasonable for preservation of pancreatic function in hemodynamically stable condition. The preservation of the spleen should be also considered in hemodynamically stable patients. Kristinsson et al. reported that cancer-free splenectomized patients had an increased risk of infections, thromboembolism, and malignancies, and the risks were increased after a long latency period (> 10 years) [8]. In particular, since trauma patients have many young people, preservation of the pancreas and spleen is desirable if possible.

Second, an ENPD catheter is also useful after surgery. External drainage facilitates the immediate evaluation of the volume and property of the drained pancreatic juice. Postoperative evaluation of MPD can be easily conducted by injecting contrast medium through the ENPD catheter, which can be replaced with EPS using a guidewire, and this facilitates changing the external drainage to the internal drainage. In several reports, external drainage was performed, although the catheter or T-tube was exteriorized from the stomach or duodenum and was pulled out through the abdominal wall [9–11]. Placement of an ENPD catheter is a significantly less invasive external drainage than the conventional procedure.

In several reports, ERP is useful not only as a diagnostic tool used for MPD injury but also as a non-operative treatment tool [1, 3, 4], and injury was treated with EPS alone. By contrast, this report first described the successful treatment with the combination of preoperative placement of an ENPD catheter and surgery. ERP should be used as a diagnostic tool when the presence of MPD disruption cannot be identified on CT scan or MRCP. On the other hand, it is necessary to keep in mind that ERP may lead to severe complications, such as pancreatitis; thus, we have to carry on ERP with close monitoring of the patient. Patients with grade I/II pancreatic injuries without MPD disruption must be cautiously followed up. Moreover, in those with MPD injury or suspected injury, ENPD catheter or EPS should be placed across the injured site. When the procedure fails, as in our case, an ENPD catheter is placed at the injury site preoperatively, and during surgery, an ENPD catheter is inserted into the distal MPD segment and the pancreas may be preserved. If such procedure cannot be carried out, pancreatic resection is considered. This strategy is indicated for hemodynamically stable patients only as it is a less invasive treatment for pancreatic injury.

## Conclusion

ERP and preoperative placement of an ENPD catheter should be well considered in the treatment of pancreatic injury in hemodynamically stable patients. The treatment strategy may allow the pancreas and spleen to be preserved.

## Data Availability

The datasets supporting the conclusions of this article are included within the article and its additional files.

## Abbreviations

AAST: American Association for the Surgery of Trauma
CT: Computed tomography
ENPD: Endoscopic nasopancreatic drainage
EPS: Endoscopic pancreatic stenting
ERP: Endoscopic retrograde pancreatography
MPD: Major pancreatic duct
MRCP: Magnetic resonance cholangiopancreatography
POD: Postoperative day
